# Whole Genome Sequence and Comparative Genomics Analysis of Multi-drug Resistant Environmental *Staphylococcus epidermidis* ST59

**DOI:** 10.1534/g3.118.200314

**Published:** 2018-04-30

**Authors:** Zhen Xu, Raju Misra, Dorota Jamrozy, Gavin K. Paterson, Ronald R. Cutler, Mark A. Holmes, Saheer Gharbia, Hermine V. Mkrtchyan

**Affiliations:** * Department of Public Health, National Demonstration Center for Experimental Preventive Medicine Education, Tianjin Medical University, Tianjin, China 300070; † School of Biological and Chemical Sciences, Queen Mary University of London, London, UK E1 4NS; ‡ Natural History Museum, Core Research Laboratories, Molecular Biology, London, UK SW7 5BD; § The Wellcome Trust Sanger Institute, Cambridge, UK CB10 1SA; ** The Royal (Dick) School of Veterinary Studies and Roslin Institute, University of Edinburgh, Easter Bush Campus, Midlothian, UK EH8 9LE; †† Department of Veterinary Medicine, University of Cambridge, Cambridge, UK CB3 0ES

**Keywords:** *Staphylococcus epidermidis*, antibiotic resistance, whole genome sequence, comparative analysis

## Abstract

*Staphylococcus epidermidis* is a major opportunistic pathogen primarily recovered from device-associated healthcare associated infections (DA-HAIs). Although *S. epidermidis* and other coagulase-negative staphylococci (CoNS) are less virulent than *Staphylococcus aureus*, these bacteria are an important reservoir of antimicrobial resistance genes and resistance-associated mobile genetic elements that can be transferred between staphylococcal species. We report a whole genome sequence of a multidrug resistant *S. epidermidis* (strain G6_2) representing multilocus sequence type (ST) 59 and isolated from an environmental sampling of a hotel room in London, UK. The genome of *S. epidermidis* G6_2 comprises of a 2408357 bp chromosome and six plasmids, with an average G+C content of 32%. The strain displayed a multi-drug resistance phenotype which was associated with carriage of 7 antibiotic resistance genes (*blaZ*, *mecA*, *msrA*, *mphC*, *fosB*, *aacA-aphD*, *tetK*) as well as resistance-conferring mutations in *fusA* and *ileS*. Antibiotic resistance genes were located on plasmids and chromosome. Comparative genomic analysis revealed that antibiotic resistance gene composition found in G6_2 was partly preserved across the ST59 lineage.

*Staphylococcus epidermidis* is a common human skin commensal, but also the most frequent pathogen among coagulase-negative staphylococci (CoNS), causing primarily device-associated healthcare associated infections (DA-HAIs). Compared with more virulent *S. aureus*, CoNS rarely produce toxins and less is known on whether the toxin genes contribute to strain virulence (Otto 2013a). *S. epidermidis* forms biofilms on medical devices and implants, from which single cells dissociate and disseminate via the bloodstream to start colonization at a different site, which might lead to sepsis, meningitis and endocarditis (Becker *et al.* 2014). In addition, *S. epidermidis* and other CoNS are believed to act as a reservoir of resistance and virulence genes for *S. aureus*, contributing to the evolution and emergence of successful clones of methicillin-resistant *S. aureus* (MRSA) (Otto 2013b).

Together with *S. aureus* and other CoNS, *S. epidermidis* accounts for 30% of hospital associated infections (Conlan *et al.* 2012). These nosocomial pathogens have developed an arsenal of strategies contributing to colonization and infection of the hosts (Becker *et al.* 2014), while often being resistant to multiple antibiotics. Emergence of antibiotic resistant bacteria has been mostly attributed to the healthcare-associated settings (Oliveira and Tomasz 2002). However, more recently, selection of antibiotic resistance has been also associated with the community which has been linked to the misuse of antibiotics (DeLeo *et al.* 2010). A typical example of this is the community-acquired MRSA (CA-MRSA) which, in addition to acquiring methicillin resistance, has gradually increased the frequency of resistance determinants similarly to hospital-acquired MRSA (HA-MRSA) (Chambers 2005). There is an increasing evidence that horizontal gene transfer between closely related species may contribute to this (Otto 2013a). Recently, Méric *et al.* showed that *S. aureus* and *S. epidermidis* share half of the genome and while homologous recombination between the two species was rare, there was an evidence of extensive MGE sharing, in particular SCC*mec*, metal resistance and SaPIn1 elements (Méric *et al.* 2015). As a result, attention is now focusing on the multidrug-resistant coagulase-negative staphylococci and their rapid spread as opportunistic pathogens particularly in relation to patients with an immuno-compromised status (Morfin-Otero *et al.* 2012). Multidrug-resistant coagulase-negative staphylococci (MDR-CoNS) are primarily recovered from healthcare-associated medical devices, ambulatory patients and healthy animals (Becker *et al.* 2014).

Molecular approaches such as pulse field gel electrophoresis and multi-locus sequence typing have been widely used to evaluate the dissemination of resistant clones of bacteria (Miragaia *et al.* 2008). Recently, complete genome sequencing of *S. epidermidis* strains have been reported, however these are limited to commensal and nosocomial strains (Conlan *et al.* 2012; Gill *et al.* 2005; Zhang *et al.* 2003). Only one study has compared whole genome sequences of four *S. epidermidis* isolated from rice seeds with that of type strain (Chaudhry and Patil 2016). To our knowledge this is the first whole genome based study looking at MDR-CoNS isolated from general public settings.

In this study, we present the genetic features of this multidrug resistant *S. epidermidis* (strain G6_2) and compare it with six *S. epidermidis* reference genomes and 133 previously published genomes of clinical *S. epidermidis*.

## Material And Methods

### Isolates analyzed in this study

Between October 2012 and April 2013, we sampled different sites in three hotels in London, UK. Permission to carry out sampling was granted by the manager/owner of each hotel and the results from each hotel were reported to each manager/owner for their information. Inanimate objects in 32 hotel rooms were sampled using COPAN dry swabs (Copan Diagnostics Inc., USA). All specimens were inoculated onto Nutrient Agar (Oxoid, Basingstoke, UK) and Mannitol Salt Agar plates (Oxoid Basingstoke, UK). These cultures were incubated aerobically at 37° for 24–72 h.

The *S. epidermidis* G6_2 was recovered from one of the hotel rooms in April 2013 in London, UK. Preliminary identification was achieved by using Matrix-assisted laser desorption ionization time-flight mass-spectroscopy (Microflex LT, MALDI-TOF-MS, Bruker Daltonics, Coventry, UK) as described previously (Mkrtchyan *et al.* 2013).

For comparative genomics analysis genomes of six *S. epidermidis* reference strains were included: RP62A (Gill *et al.* 2005), ASM1192v1), ATCC12228 (Zhang *et al.* 2003), ASM764v1), SEI (Davenport *et al.* 2014), CP009046), 949_S8 (Biswas *et al.* 2015), CP010942), PM221 (Savijoki *et al.* 2014), HG813242), and BPH 0662 (Jyh *et al.* 2016), NZ_LT571449) together with 129 *S. epidermidis* genomes derived from two previously published collections (Roach *et al.* 2015; Tewhey *et al.* 2014).

### 16S rRNA gene sequencing

Genomic DNA of *S. epidermidis* G6_2 was prepared using a Qiagen DNA extraction kit (Qiagen, Crawley, UK). 16S rRNA amplification was performed as described previously (Okazaki *et al.* 2009), PCR products were sequenced by Eurofins MWG GmBH (Ebersberg, Germany) using ABI 3730 L DNA analyzer.

### Molecular characterization of S. epidermidis G6_2

Carriage of the *mecA* gene was determined with PCR as described previously (Hanssen *et al.* 2004). SCC*mec* typing was carried out by determination of *mec* and *ccr* complexes (Kondo *et al.* 2007). Multi locus sequence tying (MLST) has been used to determine seven housekeeping genes as describe previously (Thomas *et al.* 2006). Sequence types were determined using MLST V1.8 software (https://cge.cbs.dtu.dk/services/MLST/).

### Antibiotic susceptibility testing

The antibiotic susceptibility of *S. epidermidis* G6_2 was tested against 13 antibiotics (Mast Group, Merseyside, UK) using disk diffusion methods according to BSAC guidelines (J. M. Andrews and Howe 2011). This included penicillin (1 unit), amoxicillin (10 µg), cefoxitin (10 µg), oxacillin (1 µg), cefepime (30 µg), vancomycin (5 µg), gentamicin (10 µg), streptomycin (10 µg), mupirocin (20 µg), erythromycin (15 µg), tetracycline (10 µg), fusidic acid (10 µg) and chloramphenicol (30 µg). In addition, the minimum inhibitory concentration (MIC) of the isolate to oxacillin was determined using ‘‘M.I.C. evaluators’’ (Oxoid Ltd., Basingstoke, UK).

### Whole genome sequencing, assembly and comparative genomics

Genomic DNA was extracted using the MasterPure Gram Positive DNA Purification Kit (Cambio, Dry Drayton, UK) from overnight cultures grown from single colonies in 5 ml of tryptic soy broth overnight at 37°. Illumina library preparation was carried out as described previously (Quail *et al.* 2008), and genome sequencing using Hi-Sequation 2000 performed following the manufacturer’s standard protocols (Illumina, Little Chesterfield, UK). The raw fastq data were quality trimmed using trimmomatic, (version 0.35) default settings, specifying a phred cutoff of Q20. Read quality was assessed using FastQC (Andrews 2011) and Kraken (version 0.10.5-beta) metagenomic pipeline (Wood and Salzberg 2014), including KronaTools (version 2.5) (Ondov *et al.* 2011) was used to assess library purity, that is, it was not a mixed sample and ensure the species was *S. epidermidis*. *De novo* assemblies were performed using assembler, SPAdes (version 3.5.0) (Bankevich *et al.* 2012), default PE settings, from which only contigs greater than 500 bp in length were taken for further analysis. Using the program, Andi (version 0.9.4-beta) (Haubold *et al.* 2015) the *de novo* assembled G6_2 genome along with 108 assembled Staphylococci genomes were aligned, clustered and visualized using PHYLIP (http://evolution.genetics.washington.edu/phylip.html) and FigTree (http://tree.bio.ed.ac.uk/software/figtree/). Annotations were performed using the pipeline Prokka (version 1.11) (Seemann 2014). The resultant annotated genome was used for all subsequent comparative genomic studies. Carriage of antimicrobial resistance and virulence genes was assessed using the SRST 2 software (Inouye *et al.* 2014) and the ARG-ANNOT (Gupta *et al.* 2014) and VF-DB databases (Chen *et al.* 2016). Pan-genome analysis was performed using the Roary pipeline (version 3.4.2) (Page *et al.* 2015). To reconstruct phylogenetic tree, short reads were mapped against the *S. epidermidis* ATCC12228 reference genome (Zhang *et al.* 2003), using SMALT version 0.5.8 (http://www.sanger.ac.uk/science/tools/ smalt-0). A core genome alignment was created after excluding MGE regions, variable sites associated with recombination (detected with Gubbins (Croucher *et al.* 2015) and sites with more than 5% proportion of gaps (*i.e.*, sites with an ambiguous base). A maximum likelihood (ML) phylogenetic tree was generated with RAxML v8.2.8 (Stamatakis 2014) based on generalized time reversible (GTR) model with GAMMA method of correction for among site rate variation and 100 bootstrap (BS) replications. The phylogenetic tree was annotated using Evolview (Zhang *et al.* 2012).

### Nucleotide sequence accession numbers

Reads for *S. epidermidis* G6_2 were submitted to the European Bioinformatics Institute Sequence Read Archive, accession ERR387168.

### Data availability

The authors state that all data necessary for confirming the conclusions presented in the article are represented fully within the article and its tables and figures. Supplemental material available at Figshare: https://doi.org/10.25387/g3.6133946.

## Results and Discussion

*S. epidermidis* has become a leading hospital-associated pathogen due to the increased use of medical devices (Vuong *et al.* 2004). Treatment of *S. epidermidis* infections is challenging as the bacteria are commonly resistant to methicillin and might also display multi-drug resistance phenotype, which presents a serious public health challenge (Xu *et al.* 2015) . *S. epidermidis*, represents an important reservoir of mobilizable genes that can be horizontally transferred between staphylococci species, which has likely contributed to the development of antibiotic resistance in *S. aureus* (Otto 2013a).

*S. epidermidis* G6_2 was isolated from a hotel room in London, UK in 2013, and the species were determined by MALDI-TOF MS and 16S rRNA sequencing. Initial molecular analysis revealed that the *S. epidermidis* G6_2 strain was *mecA* positive, carrying SCC*mec* type IV, and represented ST59.

A draft genome was assembled, comprising of 53 contigs (48 >= 1kb) for the isolated *S. epidermidis* G6_2 genome (Table S1; Table S2 and Figure S1). The assembly comprised of one chromosome (2408357 bp in length) and six plasmids, annotated as pG6_2_1 to pG6_2_6 (the largest, pG6_2_1, is 10570 and the smallest, pG6_2_6, is 3426 bp in length), with an average G+C content of 32.02%. It has a total (chromosome and plasmids) of 2213 predicted protein coding sequences, of which 21.5% were annotated as hypothetical proteins and 14.3% were annotated as putative functions (Table 1).

**Table 1:** Comparative general features of *S. epidermidis* G6_2 and the reference strains

Chromosome*^a^*	RP62a	ATCC 12228	SEI	949_S8	PM221	BPH 0662	G6_2
Length of sequences (bp)	2616530	2499279	2538314	2339868	2490012	2793003	2408357
G+C content	32.10%	32.10%	32.10%	32.00	32.10%	32.00%	32.02%
Protein coding region	2391	2419	2504	2119	2399	2699	2213
Ribosomal RNAs							4
16S	6	5	6	-*^b^*	6	5	1
23S	6	5	6	-*^b^*	6	5	1
5S	7	6	7	5	7	6	2
Transfer RNAs	59	60	58	56	59	59	60
Plasmids*^c^*							
Length of sequences (bp)	P1:27310	P1:4439	P1:37688	-*^b^*	P1:4439	P1:45804	P1:10570
		P2:4679			P2:11152	P2:2366	P2:4909
		P3:8007			P3:33094		P3:4588
		P4:17261			P4:58811		P4:4576
		P5:24370					P5:4271
		P6:6585					P6:3426

a Chromosome section includes: the length of the chromosome, G+C content of the chromosome, protein coding region, ribosomal RNA and transfer RNAs numbers.

b ‘-’ No data available in Genbank file. Draft assembly.

c Plasmids section includes: the length of each plasmid and the number of plasmids. P - Plasmid. Numbers - the number of plasmids.

### Phylogenetic relationship with other S. epidermidis isolates

**Figure 1:**
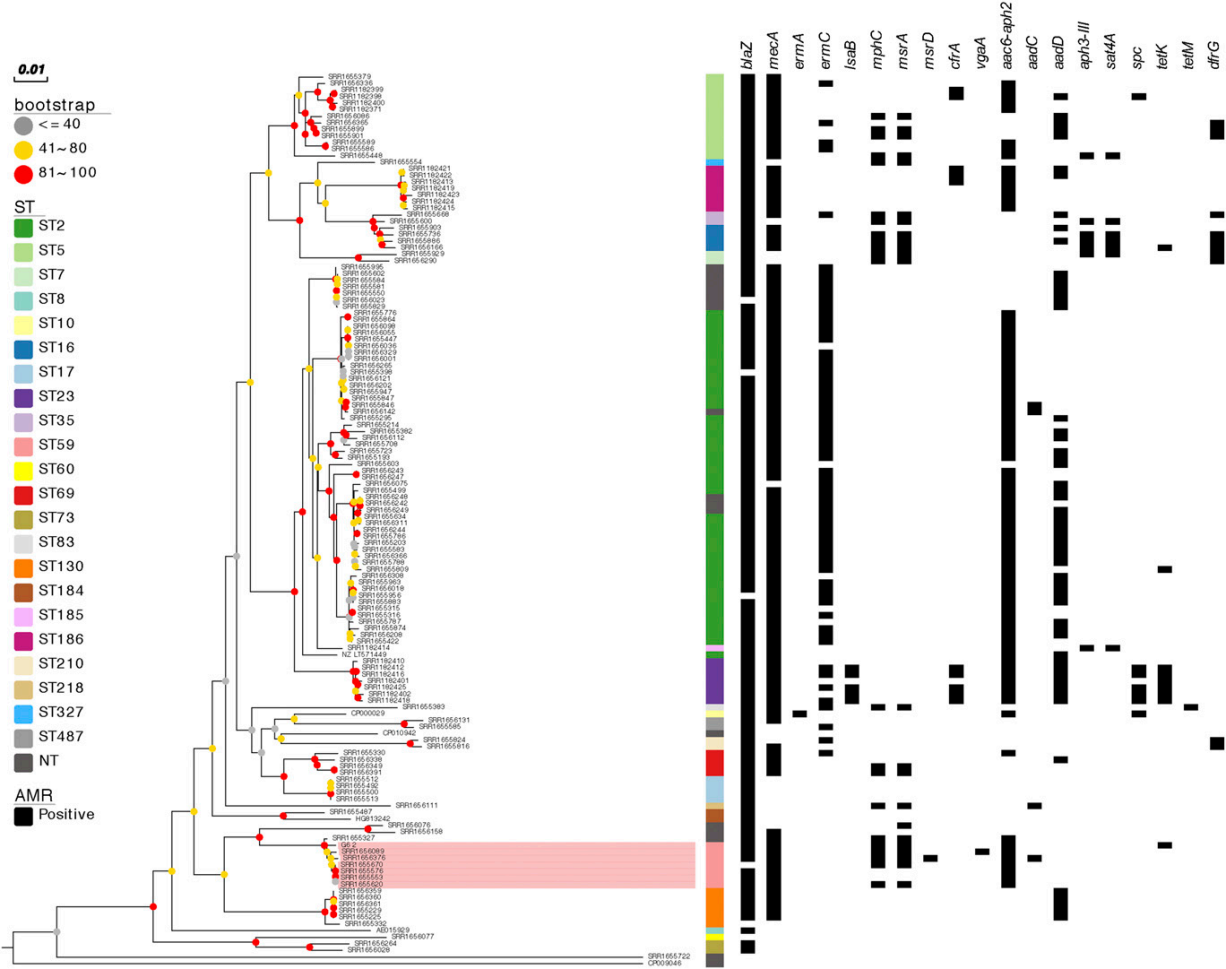
Core-genome mid-point rooted phylogenetic tree of 136 *S. epidermidis* isolates. The tree nodes are annotated with bootstrap value ranges based on 100 replicates. The tree is also annotated with the sequence type (ST) assignment and antimicrobial resistance gene (AMR) carriage. Gene names above the annotation are grouped in accordance with the corresponding antimicrobial class (beta-lactams: *blaZ*, *mecA*; macrolides, lincosamides and streptogramines: *ermA*, *ermC*, *lsaB*, *mphC*, *msrA*, *msrD*, *cfrA* and *vgaA*; aminoglycosides: *aac6-aph2*, *aadC*, *aadD*, *aph3*-III, *sat4A* and *spc*; tetracyclines: *tetK* and *tetM*; trimpethoprim: *dfrG)*. The ST59 cluster that contains the G6_2 strain is highlighted in pink.

A previously described collection of 129 whole genome-sequenced *S. epidermidis* isolates together with 6 reference strains was used to determine the phylogenetic relationship between the G6_2 strain and other *S. epidermidis lineages*. After removal of variable sequence regions corresponsing to mobile genetic elements (MGE), recombination blocks as well as sites with more than 5% proportion of gaps, the core genome alignment contained 4262 SNP sites. Seven ST59 isolates clustered and formed a distinct clade with *S. epidermidis* G6_2 (Figure 1).

### Genotypic and phenotypic characterization of antibiotic resistance

*S. epidermidis* G6_2, revealed 9 antibiotic resistance determinates across the chromosome and plasmids (Table 2). This included aminoglycoside resistance gene *aac(6’)* – *aph(2”)*, beta-lactam resistance genes *mecA* and *blaZ*, fosfomycin resistance gene *fosB*, macrolide resistance genes *mphH* and *msrA* (the latter also conferring resistance to lincosamide and streptogramin B) and tetracycline resistance gene *tet(K)*. This correlated with the results of antibiotic susceptibility testing as the strain was found resistant to 11 out of 13 antibiotics tested, demonstrating susceptibility to vancomycin and chloramphenicol only. Resistance to mupirocin and fusidic acid was associated with point mutations in chromosomally located genes, *ileS* and *fusA*, respectively. In addition to antimicrobial resistance genes, the G6_2 strain also carried plasmid-associated *qacC* gene, which encodes the multidrug resistance efflux protein and mediates resistance to biocides, and a chromosomally-inserted copper resistance operon composed of *copZ-copA-csoR* genes together with an additional copy of cobalt-zinc-cadium efflux pump gene *czcD*. The latter was distinct from the conserved chromosomal copy of *czcD* gene, and was previously identified on a number of CoNS plasmids.

**Table 2:** Genotypic and phenotypic characterization of antibiotic resistance in *S. epidermidis* G6_2

Product	Gene name	Accession number (Identity %)	Location	Function	Class of antibiotic	Antibiotics
Aminoglycoside-modifying enzymes	*aac(6’)-aph(2’’)*	M13771 (100)	plasmid	Aminoglycoside resistance	Aminoglycoside	Gentamycin streptomycin
β-lactamase	*blaZ*	AJ302698 (100)	plasmid	Beta-lactam resistance	Beta-lactam	Penicillin oxacillin Amoxcillin cefepime cefoxitin
Penicillin-binding protein 2a	*mecA*	AB505628 (100)	Chromosome	Beta-lactam resistance	
Fosfomycin resistance protein	*fosA*	ACHE01000077 (100)	Chromosome	Fosfomycin resistance	Phosphonic	Fosfomycin
Macrophage scavenger receptors	*msr(A)*	X52085 (98.98)	plasmid	Macrolide, Lincosamide and Streptogramin B resistance	Microlide	Erythromycin
Inactivating enzymes	*mph(C)*	AF167161 (100)	plasmid	Macrolide resistance	
Tetracycline efflux pump	*tet(K)*	U38428 (99.93)	plasmid	Tetracycline resistance	Tetracycline	Tetracycline
Isoleucyl RNA synthetase	*ileS*	—	—	Fusidic acid resistance	Fusidic acid	Fusidic acid
Elongation factor G	*fusA*	—	—	Monoxycarbolic resistance	Monoxycarbolic	Mupirocin

The G6_2 strain carried a 47-kb composite island composed of the SCC*mec* IV and a SCC element that contained plasmin-sensitive surface protein gene *pls*, spermidine N-acetyltransferase gene *speG* and a copper-translocating ATPase gene *copA*. The full sequence of this composite island was unique and did not match previously described reference genomes. However, the SCC*mec* IV sequence shared 99% identity with SCC*mec* IVa from various MRSA strains including the MRSA M1 isolated in Denmark (Larner-Svensson *et al.* 2013). The SCC element matched most closely the MRSA UCI62 strain representing ST5 (GenBank: CP018766). Carriage of *blaZ*, *tetK* and *qacC* genes was associated with plasmid sequences whereas other genes were inserted chromosomally. Elements carrying *tetK* and *qacC* matched previously reported *S. aureus* plasmids. Méric *et al.* showed that hospital associated *S. aureus* and *S. epidermidis* share genes involved in pathogenicity, metal toxicity resistance and antibiotic resistance. In addition they have demonstrated that high levels of recombination of genes that might be successful in healthcare settings contribute to proliferation of subpopulations of two species (Méric *et al.* 2015).

Comparison of resistance determinant distribution revealed that the *S. epidermidis* G6_2 strain shared a common antibiotic resistance gene composition with other ST59 isolates, suggesting that the particular combination of antibiotic resistance genes found in the G6_2 strain is preserved across the ST59 lineage (Figure 1). All ST59 isolates harbored *aac-aph*, *blaZ* and *mecA* genes, and majority contained *mphC* and *msrA* genes, whereas *tetK* was uniquely found in *S. epidermidis* G6_2. The G6_2 strain also shared the *qacC* plasmid with other ST59 isolates as well as the SCC*mec* IV sequence but not full SCC*mec*-SCC composite island, which was not detected in any other analyzed *S. epidermidis* genome.

### Functional genes uniquely found in S. epidermidis G6_2 compared with reference strains

Pan-genome analysis of the G6_2 strain and six *S. epidermidis* reference genomes revealed that 78 genes were unique to G6_2. After excluding genes found on plasmids, 64 chromosomally located genes were unique to G6_2 strain. This included a number of SCC*mec*- and SCC-associated genes as well as some of the chromosomally inserted resistance genes such as *mphC*, *msrA*, *copZ-copA-csoR* operon and the additional copy of *czcD* genes.

### Comparative analysis of virulence genes

Pathogenicity of *S. epidermidis* has been linked primarily with its capacity for biofilm formation. Biofilm formation occurs by initial attachment of bacteria on both biotic and abiotic surfaces, which further accumulates into multi-layered cell agglomerates. This facilitates the internalization and persistence of *S. epidermidis* species in the host cells. Strains that facilitate this feature are therefore considered more virulent (Becker *et al.* 2014). *S. epidermidis* carries a number of virulence determinants that have been associated with its ability to attach to biotic and abiotic surfaces as well as the various phases of biofilm formation. Analysis of virulence gene composition based on the VF database, revealed a number of such virulence determinants that were detected in all or majority of analyzed *S. epidermidis* isolates, including the G6_2 strain. This included the autolysin gene *atlE* (138/140), the cell wall associated fibronectin binding protein gene *ebh* (140/140), the elastin binding protein gene *ebp* (135/140), the fibrinogen binding protein genes *sdrG* (137/140) and *sdrH* (138/140), serine protease genes *sspA* (138/140) and *sspB* (138/140), lipase genes *geh* (139/140) and *lip* (138/140), and the nuclease gene *nuc* (138/140). The intercellular adhesion operon *icaADBC*, which is also associated with biofilm formation (Cramton *et al.* 1999), was variably distributed (87/140) and absent in the *S. epidermidis* G6_2 strain as well as the other ST59 isolates included in this analysis. This is in agreement with previous reports of clinical *S. epidermidis* ST59 isolates that revealed a biofilm negative phenotype (Li *et al.* 2009; Mendes *et al.* 2012; Miragaia *et al.* 2007).

In addition to the described biofilm formation-associated virulence determinants, majority of *S. epidermidis* isolates carried the hemolysin-beta gene *hlb* (136/140), which was also present in the G6_2 strain. Less common was the delta hemolysin gene *hld* (41/140), also detected in the G6_2 strain although absent in most other ST59 isolates.

In conclusion, this study is the first analysis of the genome of *S. epidermidis* isolated from the general public environment and harboring a cassette of resistance genes to an array of antimicrobials. The comparison of *S. epidermidis* G6_2 genome with clinical reference strains revealed its antibiotic resistance and virulence gene arsenal. Resistance genes were carried on both bacterial chromosome and plasmids. We established that *S. epidermidis* G6_2 harbors 12 virulence genes, and delta hemolysin gene *hld* (41/140) is known to be detected in the G6_2 strain but absent in most other ST59 isolates. In addition, 9 antibiotic resistance determinants which are responsible for the resistance to 12 antibiotics, including streptomycin, gentamicin, penicillin, oxacillin, amoxicillin, cefoxitin, cefepime, erythromycin, fosfomycin, tetracycline, fusidic acid, mupirocin, have been identified in *S. epidermidis* G6_2. Additional whole genome sequence and comparative genomics analysis are warranted to further our understanding of the origin and evaluation of multidrug resistant isolates from different ecological niches.
